# A case of complete resection of prostate stromal sarcoma after neoadjuvant chemotherapy

**DOI:** 10.1002/iju5.12596

**Published:** 2023-05-14

**Authors:** Naoto Hodotsuka, Yasutomo Suzuki, Kyota Suzuki, Yuichiro Honda, Shuma Endo, Eigo Kuribayashi, Kotaro Obayashi, Tadaaki Minowa, Tsutomu Hatori, Yukihiro Kondo

**Affiliations:** ^1^ Department of Urology Nippon Medical School Chiba Hokusoh Hospital Chiba Japan; ^2^ Department of Urology Koto Hospital Tokyo Japan; ^3^ Department of Pathology Nippon Medical School Chiba Hokusoh Hospital Chiba Japan; ^4^ Department of Urology Nippon Medical School Hospital Tokyo Japan

**Keywords:** docetaxel, gemcitabine, neoadjuvant chemotherapy, prostate, stromal sarcoma

## Abstract

**Introduction:**

Prostatic stromal sarcoma is an extremely rare malignancy of the prostate with a poor prognosis.

**Case presentation:**

A 65‐year‐old man presented with dyschezia, and computed tomography showed a large prostate mass. The diagnosis was prostate stromal sarcoma by transrectal needle biopsy. Magnetic resonance imaging suggested rectal infiltration. The patient underwent 4 courses of neoadjuvant chemotherapy with gemcitabine and docetaxel hydrate followed by total pelvic exenteration.

**Conclusion:**

No recurrence has occurred at 5 years after the surgery. This is the first report of complete resection in prostate stromal sarcoma after neoadjuvant chemotherapy with gemcitabine and docetaxel hydrate.

**Abbreviations & Acronyms**
AC: adjuvant chemotherapy
ACT: actinomycin
AWD: alive with disease
CDDP: cisplatin
CPT‐11: irinotecan
CT: computed tomography
CTh: chemotherapy
DOD: dead of disease
DOXO: doxorubicin
DR: distant recurrence
DTX: docetaxel hydrate
Dx: diagnosis
ER: estrogen receptors
GEM: gemcitabine
IFO: ifosfamide
LR: local recurrence
MMC: mitomycin C
MRI: magnetic resonance imaging
n.r.: not reported
NAC: neoadjuvant chemotherapy
NCI CTCAE: National Cancer Institute Common Terminology Criteria for Adverse Events
NED: no evidence of disease
PgR: progesterone receptors
PIRA: pirarubicin
PR: partial response
PSPUMP: proliferation of uncertain malignant potential
PSS: prostate stromal sarcoma
RCP: radical cystoprostatectomy
RP: radical prostatectomy
RT: radiotherapy
SD: stable disease
STUMP: stromal tumors of uncertain malignant potential
VCR: vincristine
Vp‐16: etoposide

**Keynote message**

PSS is an extremely rare malignancy with a poor prognosis. Surgical resection is considered the treatment of choice as the effectiveness of NAC is unknown. Here, we report a case of PSS, in which the tumor was completely resected after NAC with GEM and DTX.

## Introduction

PSS is an extremely rare malignancy of the prostate with a poor prognosis. In 1998, PSS was classified into 2 types: PSS and STUMP. Surgical resection is the treatment of choice, as the effectiveness of NAC is unknown.^1^ Here, we report a case of PSS, in which the tumor was completely resected after NAC with GEM and DTX.

## Case report

A 65‐year‐old man presented with dyschezia and was admitted to a local hospital in Thailand.

**Fig. 1:**
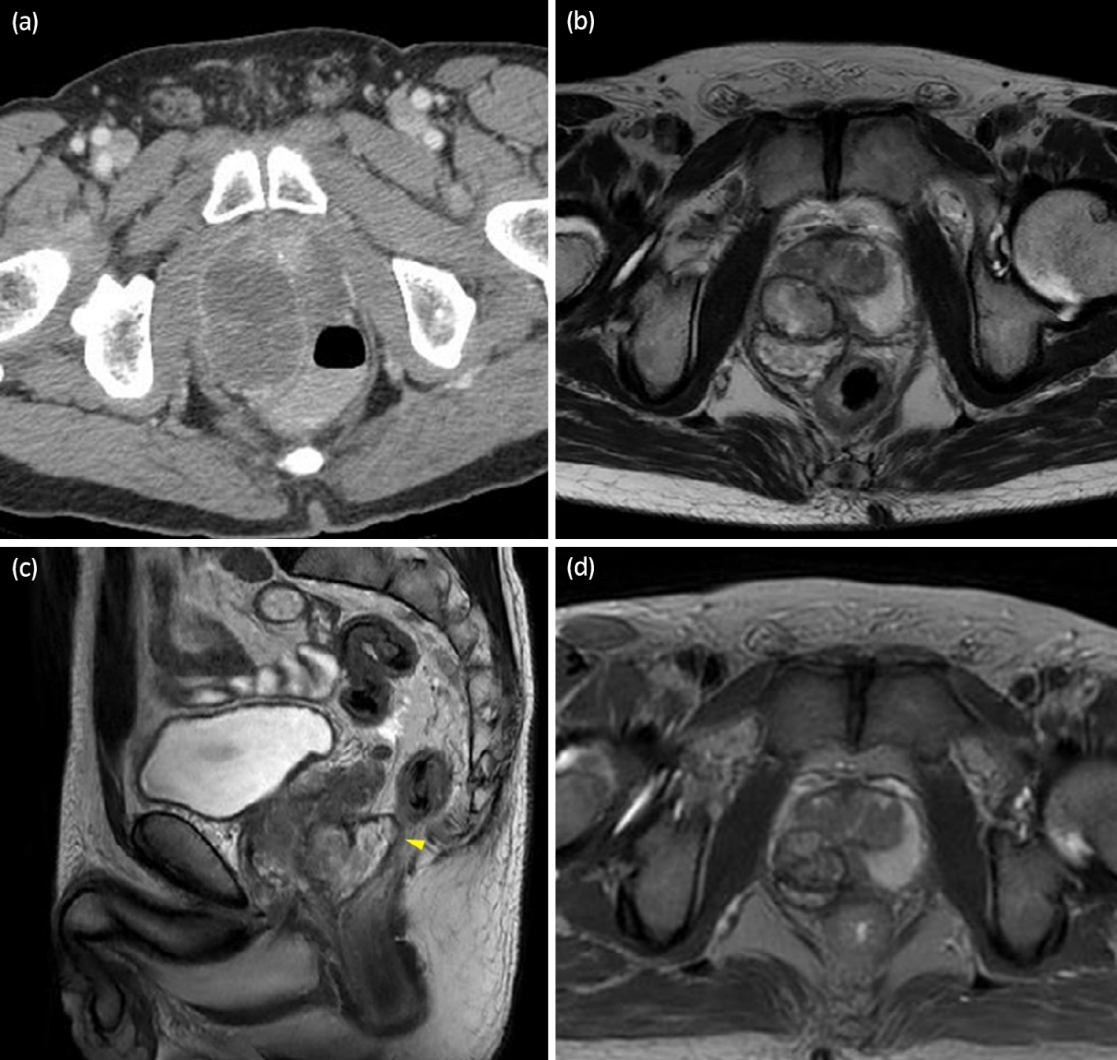
(a) Contrast‐enhanced CT shows a 62 × 40 mm^2^ mass in the right prostatic lobe. (b, c) MRI after two courses of CTh shows a 48% reduction in tumor size and suggests rectal invasion (yellow arrowhead). (d) MRI after four courses of CTh shows a 75% reduction in tumor size from the initial examination.

Rectal examination indicated a prostate mass, and contrast‐enhanced CT showed a contrast‐enhanced, 62 × 40 mm^2^ mass with rectal invasion, mainly in the right lobe of the prostate (Fig. 1a). The PSA value was 1.95 ng/ml (normal range at <4.0 ng/ml).

Prostate biopsy showed a high concentration of nuclei, necrosis, and small atypical cells, as well as increased nuclear fragmentation in the right lobe. Staining was positive for vimentin, negative for CD34, PSA, desmin, cytokeratin, and CD117, and highly positive for Ki‐67 (80%), indicating PSS. Whole‐body CT showed no lymph nodes or distant metastases.

The patient was diagnosed with PSS.

Chemotherapy with GEM 1 g/m^2^ (days 1, 8) and DTX 70 mg/m^2^ (day 1) was administered. After two courses of CTh, MRI showed that the volume of the mass was reduced by 48% to 36 × 36 mm, indicating a PR to the CTh (Fig. 1b,c). T2‐weighted MRI showed possible invasion of the rectal mucosa and serosa. After undergoing one more course of CTh, the patient returned to Japan for further treatment at our hospital.

Physical examination, all other routine blood tests, and urine tests were normal. Transrectal prostate biopsy was performed to evaluate the response to CTh. On pathological examination, coagulative necrosis of the tumor tissue, with surrounding fibrosis, histiocytic infiltration and hemosiderin deposition were observed, suggesting the effects of CTh. However, the histology was difficult to determine.

Based on the tumor shrinkage and the results of the prostate biopsy at our hospital, CTh with GEM and DTX was considered to be effective.

Another course was performed following the regimen for endometrial cancer in our hospital, a combination of GEM (900 mg/m^2^; days 1, 8) and DTX (75 mg/m^2^;days 1). Neutropenia [grade4 by NCI CTCAE] occurred on days 13–16 of treatment. MRI showed that the tumor had reduced by 75% from the initial examination (Fig. 1d). Total pelvic exenteration, an ileal conduit, and colostomy were planned.

**Fig. 2:**
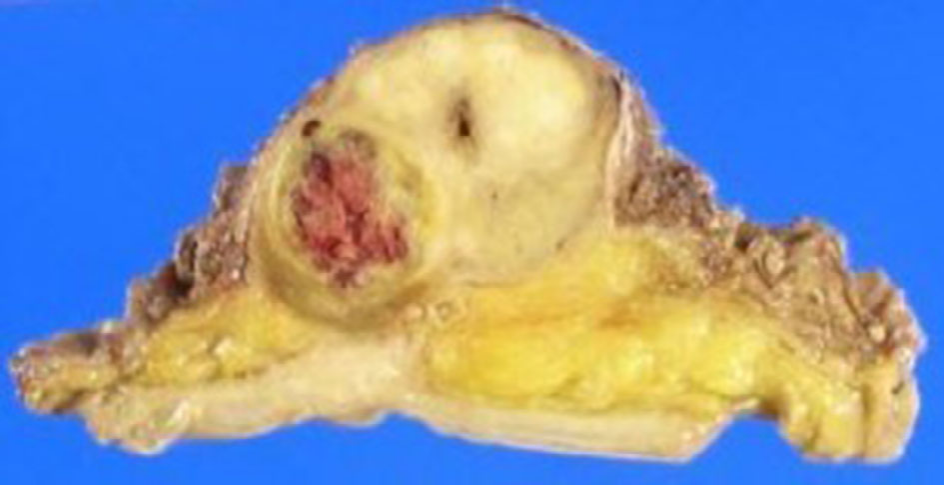
Macroscopically, the right lobe of the resected prostate shows a 2.7 × 2.3 cm^3^, yellowish‐white, smooth‐surfaced ampullary area with necrosis and hemorrhage.

**Fig. 3:**
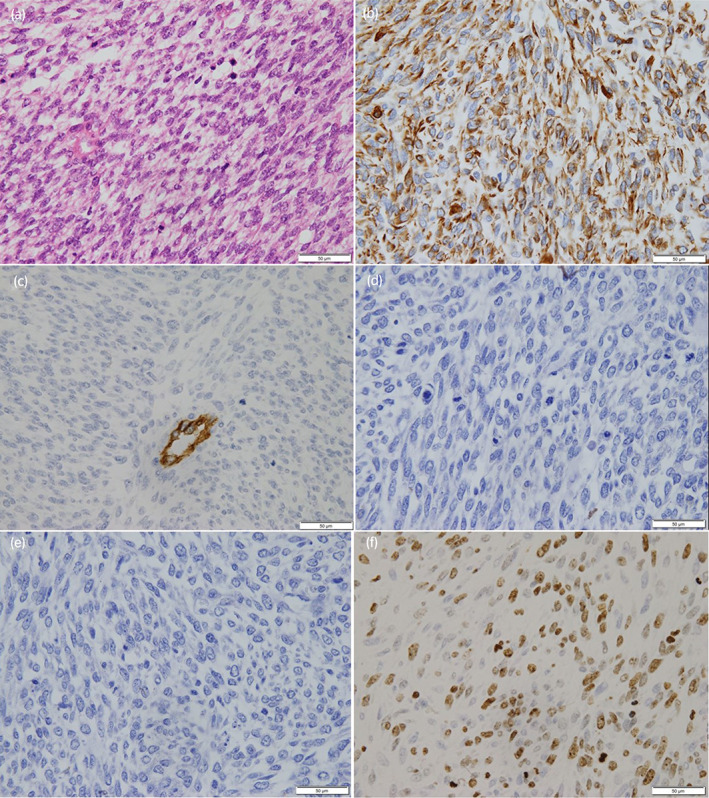
Histopathology of the prostatic stromal sarcoma. Spindle‐shaped cells are observed with an increased nucleocytoplasmic ratio. (a) Immunohistochemistry shows that the tumor is positive for vimentin (b), but negative for CD34, PgR, and ER (c–e). (f)The percentage of Ki‐67 positive cells was 50%.

After an incision in the middle lower abdomen, we observed no adhesions to surrounding organs, but the border between the tumor and rectum was indistinct. Since the length of the rectum from the pelvic floor to the tumor was found to be more than 3 cm, the anus was preserved, and a temporary small bowel stoma was created with combined resection of the rectum for a margin. Macroscopically, the right lobe of the prostate showed a 2.7 × 2.3 cm^2^, yellowish‐white, smooth‐surfaced ampullary area with necrosis and hemorrhage (Fig. 2). Histopathological findings showed necrotic tissue in the right lobe of the prostate with a small tumor component in the center. There was complex growth of spindle‐shaped cells with round to oval nuclei and narrow cytoplasm (Fig. 3a). Immunohistochemically, the tumor cells were negative for desmin, CD34, C‐kit, PgR, and ER, indicating sarcoma (Fig. 3b–e). The percentage of Ki‐67 positive cells was 50% (Fig. 3f). Vitrification and fibrosis extended into the surrounding fat, suggesting that there may have been extraprostatic invasion prior to treatment. Based on the histopathological findings, the final Dx was PSS.

The patient was discharged on postoperative day 39. The small bowel stoma was closed 126 days after total pelvic organ resection, and there was no sign of recurrence or metastasis at 5 years after surgery.

## Discussion

The main histological types of prostate sarcoma in adults are leiomyosarcoma and rhabdomyosarcoma, whereas PSS is a rarer histological type. Approximately 30 cases of PSS have been reported.^1^, ^2^ PSS was reported previously using various names, such as phyllodes tumor; however, in 1998, cases of PSS where considered either prostatic stromal PSPUMP or PSS according to the degree of cellularity, mitotic figures, necrosis, and stromal overgrowth for the purpose of understanding biological prognosis and guiding treatment.^3^ The WHO has since adopted the names STUMP and PSS.^4^

Analysis of the PSS reports indicated that patients were 22 to 65 years old (median age, 38 years). Although most initial symptoms of PSS are dysuria, hematuria, and urinary retention, PSS has no specific clinical symptoms.^2^ The most common metastatic site is the lung.^1^

There are no specific imaging findings for PSS; however, in the present case, MRI detected rectal invasion. PSS may not increase PSA levels, which were normal in the present case.^2^

Compared to STUMP, PSS shows greater cellularity, mitosis, necrosis and stromal overgrowth.^5^

While immunohistochemistry can help diagnose PSS, it does not provide a definitive Dx.^2^ PSS is generally vimentin, CD34, and PgR positive, and negative for ER, but CD34 and PgR were negative in this case.^1^, ^2^ Most cases show an increased Ki‐67 labeling index, with the present case showing an 80% increase.^1^

The treatment of PSS is mainly surgical resection, with extended resection selected according to the degree of tumor growth.^1^ Complete excision is recommended because PSS may rapidly develop LR or distant metastasis after incomplete excision.^5^ This case achieved long‐term survival with an extended resection for tumor control.

It is not known whether radiation therapy or CTh can prolong survival.^1^ Median survival is <15 months, and 10% of patients have a survival rate of >3 years.^6^ Eleven reports of CTh for PSS describe the regimens and outcomes, including the present case.^1^, ^7^, ^8^, ^9^, ^10^, ^11^, ^12^, ^13^, ^14^, ^15^ The reports are summarized in Table 1. Doxorubicin and IFO were the most commonly selected regimens.^8^, ^9^ The regimen with GEM and DTX was reported to be useful in a Phase II study for nongastrointestinal stromal sarcomas.^16^ Although the use of GEM and DTX for PSS has been reported only in the present case, it showed a clear tumor reduction effect of 75%, and surgical resection was performed at an appropriate time, thus avoiding permanent colostomy and achieving both cancer control and quality of life with a good long‐term prognosis. Results suggest that extended resection after NAC may be a treatment option for locally advanced PSS if histological Dx is obtained before surgery.

**Table 1:** Treatment, response to CTh and outcomes in 11 patients with prostatic stromal sarcoma

	Ref. (year)	Age	Tumor extension	Treatment first line	Second line	Drugs (cycles)	Response to CT	Outcome
1	11 (2001)	36	Prostate extending to neck of bladder	RP	NAC + RT (70 Gy/35f)	DOXO + IFO (6)	PR	LR, DOD 20 months after Dx
2	8 (2002)	35	Prostate, pelvic wall	RP + RT (60 Gy)	NAC + lobectomy	DOXO + IFO (5)	PR	DR (lung), NED 5 months after lobectomy
3	12 (2003)	78	Prostate extending to bladder, abdominal walls	RP + AC		VCR + MMCO + DOXO + CDDP (6)	n.r.	DR (liver), DOD 6 months after CTh
4	7 (2006)	19	Prostate	RP	NAC + RT (49.2 Gy)	VP‐16 + IFO + CDDP (4)	PR	NED 48 months after CTh and RT
5	13 (2007)	52	Prostate	RCP	NAC + metastatectomy	CDDP + PIRA + IFO (3)	n.r.	LR, AWD 12 months after metastatectomy
6	14 (2010)	31	Prostate	NAC + RP	Cystectomy	DOXO + IFO (2)	SD	LR, DOD 3 months after cystectomy
7	15 (2010)	34	Prostate	RP + RT (60 Gy)	Lung metastatectomy + palliative RT and CTh	DOXO + IFO (1)	PR	LR, DR (lung), DOD 25 months after Dx
8	16 (2012)	63	Prostate extending to bladder	NAC + total pelvic exenteration		CDDP + CPT‐11 (1)	n.r.	NED 16 months after RCP
9	9 (2014)	14	Prostate extending to bladder, seminal vesicles, lymph nodes, bone	NAC + RT (50 Gy) + RCP		IFO + VCR + ACT + DOXO (9)	PR	DOD 15 months after Dx
10	2 (2020)	40	Prostate extending to the seminal vesicles and the rectal muscularis propria.	Total pelvic exenteration + AC		DOXO + IFO (4)	n.r.	NED 10 months after RCP
11	Present case	65	Prostate extended to rectum	NAC + total pelvic exenteration		DTX + GEM (4)	PR	NED 5y after RCP

## Conclusion

In the present case of PSS, NAC using GEM and DTX and total pelvic exenteration resulted in survival without recurrence at 5 years after surgery, suggesting the effectiveness of multidisciplinary treatment for PSS.

## Author contributions

Naoto Hodotsuka: Writing – original draft. Yasutomo Suzuki: Supervision. Kyota Suzuki: Writing – review and editing. Yuichiro Honda: Writing – review and editing. Shuma Endo: Writing – review and editing. Eigo Kuribayashi: Writing – review and editing. Kotaro Obayashi: Writing – review and editing. Tadaaki Minowa: Writing – review and editing. Tsutomu Hatori: Visualization. Yukihiro Kondo: Supervision.

## Conflict of interest

The authors declare no conflicts of interests.

## Approval of research protocol by an Institutional Reviewer Board

Not applicable.

## Informed consent

The patient provided informed consent for publication of this case report.

## Registry and the Registration No. of the study/trial

Not applicable.
